# Microbial metabolism of transparent exopolymer particles during the summer months along a eutrophic estuary system

**DOI:** 10.3389/fmicb.2015.00403

**Published:** 2015-05-12

**Authors:** Edo Bar-Zeev, Eyal Rahav

**Affiliations:** ^1^Department of Chemical and Environmental Engineering, Yale UniversityNew Haven, CT, USA; ^2^Israel Oceanographic and Limnological Research, National Institute of OceanographyHaifa, Israel

**Keywords:** heterotrophic bacteria, TEP, estuary, ectoenzyme, β-glucosidase, polysaccharides, BGE, BCD

## Abstract

This study explores the role of transparent exopolymer particles (TEP) as an additional carbon source for heterotrophic microbial activity in the eutrophic Qishon estuary. From the coastal station and upstream the estuary; TEP concentrations, β-glucosidase activity, bacterial production and abundance have gradually increased. TEP were often found as bio-aggregates, scaffolding algae, detritus matter and bacteria that likely formed “hotspots” for enhance microbial activity. To further demonstrate the link between TEP and heterotrophic bacterial activity, confined incubations with ambient and polysaccharide-enriched estuary water were carried out. Following polysaccharide addition, elevated (~50%) β-glucosidase activity rates were observed, leading to TEP hydrolysis. This newly formed bioavailable carbon resulted in significantly higher growth rates, with up to a 5-fold increase in heterotrophic bacterial biomass, comprising mostly high nucleic acid content bacteria. Taking together the findings from this research, we conclude that even in highly eutrophic environments heterotrophic bacteria may still be carbon limited. Further, TEP as a polysaccharide matrix can act as a metabolic surrogate, adding fresh bioavailable carbon through tight associations with bacteria in eutrophic ecosystems such as the Qishon estuary.

## Introduction

Transparent exopolymer particles (TEP) are planktonic, acidic-polysaccharide hydrogels that are ubiquitous in various marine and fresh water environments (Passow, 2002; Bar-Zeev et al., 2015). TEP are clear and therefore only detected by specific stains such as alcian blue (Alldredge et al., 1993; Decho and Lopez, 1993). In the last two decades, TEP were shown to mediate diverse biochemical cycles in aquatic environments; TEP promote aggregate formation and sedimentation (Passow et al., 2001; Simon et al., 2002; Engel, 2004) by providing a scaffold for marine or lake “snow” (Mari and Kirboe, 1996; Grossart et al., 1997, 2006; Berman and Viner-mozzini, 2001). TEP may also serve as surface and substrate for microbes, forming “hotspots” of intense microbial activity (Azam, 1998; Simon et al., 2002; Azam and Malfatti, 2007), thus fueling the microbial loop and impacting food web structure in various ecosystems (Simon et al., 2002; Beauvais et al., 2006; de La Rocha and Passow, 2007).

Estuary systems are dynamic, semi-enclosed coastal environments where open seawater is partly diluted by fresh river water (Pritchard, 1967; Elliott and McLusky, 2002). Most estuaries worldwide are turbid and highly productive due to constant allochthonous nutrient inputs (Pinckney et al., 2001). Convergence of estuaries and open seawater often result in some of the steepest physiochemical gradients, including light penetration and oxygen concentration (Malone et al., 1996; Hall and Pearl, 2011).

The highly stratified Qishon estuary at the northern coast of Israel flows into the southeastern Mediterranean Sea, producing a steep eutrophic to oligotrophic gradient (Herut et al., 1999; Eliani-Russak et al., 2013). In the summer season, the high air temperature, low flows (0.02–0.2 m^3^ s^−1^) (Vachtman et al., 2013) and high nutrient load (as nitrate, ~240 tons per year) produce a stratified, nutrient-rich 7 km long estuary (Eliani-Russak et al., 2013). The estuary bottom layer is constantly saline (34.1–39.9) and exhibit high nutrient concentrations (1–85 μM NO_3_ and 0–9.3 μM PO_4_) throughout a spatial gradient from inland and toward the coast (Eliani-Russak et al., 2013).

In estuary ecosystems, TEP were found to play a significant role in organic matter cycling through degradation, aggregation and sedimentation processes (Simon et al., 2002; Barrera-Alba et al., 2009; Wetz et al., 2009; Mari et al., 2012; Sun et al., 2012). Hydrodynamic conditions, governed mainly by winds and tides, were reported to play a key role in TEP cycling between estuary and coastal environments (Mari et al., 2012; Sun et al., 2012). Fluvial cations passing through the estuary may also alter TEP formation (Simon et al., 2002; Wetz et al., 2009; Sun et al., 2012). In addition to its physicochemical roles in estuary systems, TEP were also suggested to act as vector for different types of pathogens (Lyons et al., 2007; Wetz et al., 2009).

In this study, we focus mainly on the metabolic feedback loops between TEP and the heterotrophic microbial community at a eutrophic estuary system. To do so, we sampled three stations along the Qishon estuary system, while conducting confined-laboratory incubation experiments. Our results shed new light on carbon limitation and polysaccharide metabolism by heterotrophic bacteria, highlighting the importance of TEP as a carbon source in a highly eutrophic environment.

## Materials and methods

### Sampling strategies and bioassay designs

**Figure 1:**
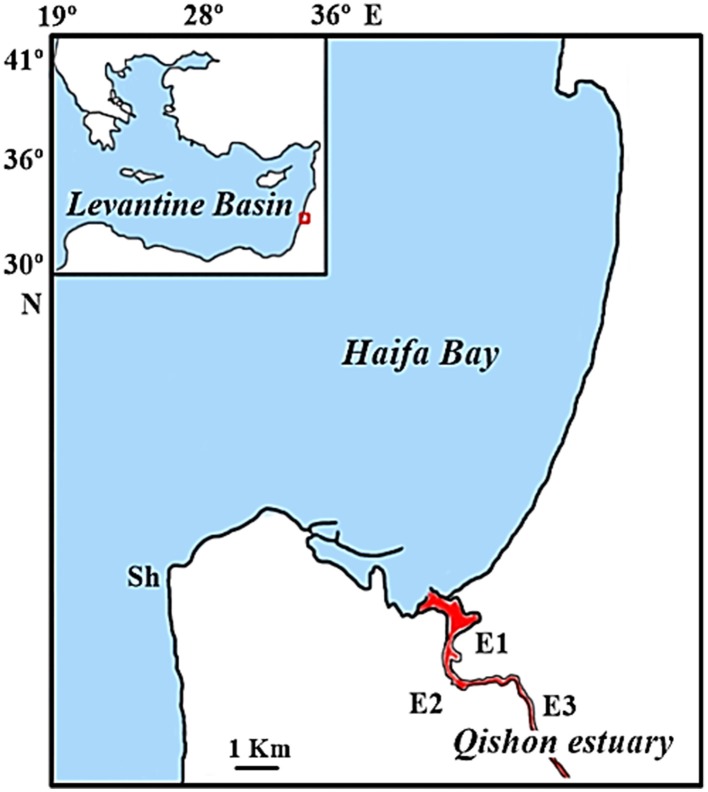
**Map of the station locations: E1–E3 are situated along the Qishon estuary and station Sh is located off the Israeli coast**.

Four stations were sampled; three along the Qishon estuary (E1; Maagan, E2; Yulius and E3; Histadrut) and one reference coastal station (Sh) (Figure 1, Table 1) during the summer months (September 2013 and June to August 2014). Temperature, salinity, dissolved oxygen (DO) and turbidity were measured in each sampling site throughout the water column using an YSI (Model 6600) profiler system. Near surface (~20 cm deep) and bottom water samples were collected in each sampling location using a 3.1 L Niskin bottle. Water samples were analyzed for TEP, bacterial production and abundance, as well as β-glucosidase activity, dissolved inorganic nutrients and chlorophyll *a* (Chl *a*).

**Table 1:** **The physicochemical characteristics of the coastal (Sh) and Qishon estuary (E1–E3) stations during summer months of 2013 (Sep.) and 2014(Jun., Jul. and Aug.)**.

**Parameter**	**Units**	**Coastal sta**.	**Estuary sta**.					
		**(Sh)**	**(E1)**		**(E2)**		**(E3)**	
		**Surface**	**Surface**	**Bottom**	**Surface**	**Bottom**	**Surface**	**Bottom**
Latitude	N	32°49′34	32°48′16		32°48′05		32°48′06	
Longitude	E	34°57′20	35°01′51		35°02′19		35°02′43	
Sampling depth	m	0.1–0.2	0.1–0.2	2–3	0.1–0.2	2	0.1–0.2	3–4
Temperature	°C	27 ± 2.6	27 ± 1.6	26 ± 2.8	28 ± 1.1	27 ± 2.3	27 ± 2.1	27 ± 2.6
Salinity		39 ± 0.4	29 ± 4.3	39 ± 0.3	20 ± 4.8	39 ± 0.4	18 ± 3.2	36 ± 0.4
pH		8.2	8.1 ± 0.1	8.2	7.9 ± 0.1	8.1 ± 0.1	7.7 ± 0.2	7.8 ± 0.1
DO	mg L^−1^	8.3 ± 0.1	6.6 ± 1.9	3.5 ± 0.8	6.3 ± 0.2	5.2 ± 2.1	4.7 ± 0.8	0.3 ± 0.1
Turbidity	NTU	1.5 ± 1.7	5.6 ± 1.5	22 ± 9.4	12 ± 3.6	41 ± 6.1	7.7 ± 0.3	23 ± 3.7
NO_2_+NO^*^_3_	μM	0.2 ± 0.1	487	65 ± 62	313 ± 159	132 ± 123	954	255 ± 108
NH^*^_4_	μM	0.8 ± 0.2	28.0	6.7 ± 6.3	74 ± 32	18 ± 18	304	55 ± 43
PO^*^_4_	μM	0.02 ± 0.01	3.5	3.7 ± 2.7	12.6 ± 2.5	5.5 ± 2.2	5.6	11.9 ± 2.3

*Values are the averages and their standard deviation represents four sampling dates*.

* *Dissolved inorganic nitrogen and phosphorus were referred as DIN and DIP in the text respectively*.

To elucidate the role of TEP as a labile carbon source for heterotrophic bacteria, we conducted two bottle incubation experiments simulating high TEP scenarios during June and August 2014. The incubation bottles were pre-washed with 10% hydrochloric acid, rinsed three times with Milli-Q water followed by three times with ambient water. At each sampling date, bottles were filled with water samples (1 L) collected from the surface and bottom of the three estuary stations (*n* = 9). Samples were than pre-filtered (1 μm filter, PALL co.) to remove most of the particulate matter, including large TEP, micro algae, filamentous cyanobacteria and grazers, while retaining planktonic bacteria. The pre-filtered water was then supplemented with gum-xanthan (GX, Sigma G1253); a commercial pure polysaccharide secreted by the bacterium *Xanthomonas campestris* (Passow and Alldredge, 1995). Gum-xanthan additions were prepared by dissolving 5 mg of GX in deionized water (100 mL) and homogenized (Thomas Scientific Model D1000) for 15 min to form concentrated small-suspended particles (Rahav et al., 2013). GX additions yielded TEP concentrations ranging from 1532 to 1648 μg GX L^−1^, which were ~8-fold higher relative to the pre-filtered, un-amended controls. The pre-filtered control and GX supplemented bottles were then incubated for 2 days under ambient temperature (25°C) in complete darkness to minimize primary production through photosynthesis. Subsamples were collected and analyzed for bacterial production, respiration and abundance, β-glucosidase activity, TEP and Chl *a* at the beginning (T_0_) and at the end (T_2d_) of the microcosm experiment. Bottles were thoroughly shaken before collecting the subsamples to resuspend any biofouling/fouling that may developed on the polycarbonate walls and aggregates that could have sedimented during the incubations.

### Dissolved inorganic nutrients

Water samples were collected in 15 mL acid-washed plastic scintillation vials. Nutrient concentrations were determined using a segmented flow Seal Analytical AA-3 system (Krom et al., 1991; Kress and Herut, 2001). The limits of quantitation of nitrate + nitrite, ammonium and phosphorus measurements were 0.19 μM, 0.15 μM and 0.02 μM, respectively. The limits of detection (twice the standard deviation of the blank) were 0.08 μM for nitrate + nitrite, 0.06 μM for ammonium and 0.008 μM for phosphorus.

### Chlorophyll *a* (Chl *a*) biomass as algal proxy

Water samples (150 mL) were passed through 0.7 μm glass fiber filters (Whatman, Lot: 1825025) and stored at −20°C, protected from light. Samples were extracted in 5 mL of 90% acetone overnight at 4°C in the dark. Chl *a* concentrations were determined using a luminescence Trilogy® fluorometer (7200-000) with a 436 nm excitation filter and a 680 nm emission filter (Holm-Hansen et al., 1965).

### Heterotrophic bacterial abundance (BA) and specific growth rates

Water samples (1.8 mL) were fixed with 6 μL of 50% glutaraldehyde (Sigma, G7651), incubated in room temperature for 10 min, flash-frozen in liquid nitrogen and stored at −80°C until further analyses. Prior to counting, the samples were thawed at 37°C for 2 min, stained with 1:10^5^ vol:vol nucleic acid SYTO ®9 (Life technologies S-7580) in the dark for 10 min and analyzed by an Attune® acoustic focusing flow cytometer (Applied Biosystems). Samples were excited with Argon lasers (488 and 405 nm) with a flow rate of 25 μL min^−1^. Heterotrophic bacteria were specifically enumerated by subtracting autotrophic bacteria (detected by Chlorophyll *a* auto-fluorescence) from the total bacterial abundance. One-micron beads (Polysciences) were used as size calibration standard. Heterotrophic bacterial abundance was converted to carbon biomass using a factor of 20 fg C per heterotrophic bacterial cell (Lee and Fuhrman, 1987). High and low nucleic acid content bacteria (HNA and LNA respectively) were differentiated by side scattering (SSC) and SYTO 9 fluorescence (488 nm excitation) that was detected by a 530/30 nm band pass filter.

Heterotrophic bacterial (HB) growth rates (k) were calculated according to the following equation:

(1) $$k_{day}=\frac{ln\;(BA_{T2}/BA_{T0})}{T_{2}-T_{0}}$$

To calculate k, heterotrophic BA was enumerated at the begging (T_0_) and the end (T_2days_) of the bottle incubations.

### Bacterial production rates (BP)

BP was measured using the [4,5-^3^H]-leucine incorporation method (Simon et al., 1990). Briefly, three aliquots (1.7 mL each) from each sample were incubated with 100 nmol L^−1^ of [4,5-^3^H]-leucine (Perkin Elmer USA, Lot: 1804258) for 4 h in room temperature in the dark. Triplicate trichloroacetic acid (TCA) inactivated samples served as controls. The incubations were terminated with 100 μL of cold (4°C) TCA (100%), followed by centrifugation (Smith and Azam, 1992) to form a pellet. After removing the supernatant and adding 1 mL of scintillation cocktail (Ultima-Gold, Quick-Safe A) to each tube, the samples were counted using a TRI-CARB 2100 TR (Packard TRI-CARB 2100 TR) liquid scintillation counter. Leucine incorporation was converted into carbon assimilation by a conservative factor of 3.1 kg C mol^−1^ with an isotope dilution factor of 2.0 (Simon and Azam, 1989).

### Dissolved oxygen (DO) and bacterial respiration rates (BR)

Oxygen was measured using the Winkler titration procedure (Carpenter, 1965). Briefly, water was immediately fixed with MnSO_4_ and KI + NaOH and sealed without headspace in 300 mL Winkler bottles (Wheaton® 227497-11). H_2_SO_4_ was later added, and samples were titrated with Na_2_S_2_O_3_ using a Metrohm 785 DMP titrino auto-burette and double platinum electrode (end-point titration precision, ± 1 μmol L^−1^) similarly to Kress et al. (2014).

Respiration rates were determined by the following equation:

(2) $$Respiration=\frac{DO_{\left(T0\;dark\right)}-DO_{\left(T48\;dark\right)}}{48h}$$

Where *DO_(T0 dark)_* is the initial dissolved oxygen concentration and *DO _(T48 dark)_* is the dissolved oxygen concentration after 48 h incubation. We assumed that in all bottle incubations bacterial respiration (BR) accounted for ~90% of the dark respiration of the entire microbial community (see result and discussion for further details).

### Bacterial carbon demand (BCD) and bacterial growth efficiency (BGE)

BCD was defined as the sum of carbon assimilation measured by bacterial production (BP) and carbon oxidation determined through heterotrophic microbial respiration (BR). Oxygen respiration was converted into carbon consumption assuming a respiratory quotient (RQ) of 1 (del Giorgio and Cole, 1998; Anesio et al., 2003; Smith and Prairie, 2004):

(3) BCD=BP+BR

BGE was calculated as follows:

(4) $$BGE\;(\%)=\frac{BP}{BP+BR}\;x100$$

### TEP concentrations and visualization with associated bacteria

Water samples (100 mL) were gently (<150 mbar) filtered through a 0.4 μm polycarbonate filter (GE Water & Process Technologies). Filters were then stained with a solution of 0.02% alcian blue (AB), 0.06% acetic acid (pH of 2.5), and the excess dye was removed by a quick deionized water rinse. Filters were than immersed in sulfuric acid (80%) for 2 h, and the absorbance (787 nm) was measured spectrophotometrically (Thermo GENESYTM). AB dye was calibrated using GX as a purified polysaccharide (Passow and Alldredge, 1995). A factor of 0.74 was used to convert from GX equivalents to carbon (Engel and Passow, 2001). This conversion factor was used as proxy, since TEP chemical composition is likely to change between different marine and fresh water environments.

To visualize TEP with bacterial associations, samples (100 mL) were filtered gently (<150 mbar) onto 0.4 μm polycarbonate filters (GE Water and Process Technologies) and stained with 0.2 μm pre-filtered AB solution and 30 μL SYTO®9 (250 μg mL^−1^) for TEP and bacterial identification, respectively (Bar-Zeev et al., 2011). After 7 min incubation in the dark, filters were washed with deionized water (5 mL) and mounted on a coated Cytoclear (Clearing Slides, GE Osmonics Labstore) slide. TEP images were taken with bright-field (Nomarski) illumination; bacteria were visualized with an epifluorescent microscope (Olympus BX50 microscope) equipped with FITC filter (excitation: 488 nm; emission: 520 nm). The detection of Chl *a* florescence was examined by a specified filter set (ex: 450 nm, em: 680 nm).

### β -glucosidase activity rates (β-glu)

The hydrolytic activity of β-glucosidase was determined by cleavage rates analysis of a conjugated fluorogenic substrate, 4-methylumbelliferyl (MUF)-β-D-glucopyranoside (Sigma M3633) (Hoppe, 1993; Luna et al., 2012). Briefly, the substrate (final concentration of 50 μM) was added to a 1 mL water sample in triplicate and incubated in the dark at ambient temperature (25°C) for 24 h. The increase in fluorescence was measured at 365 nm excitation and 455 nm emission (GloMax®-Multi Detection System E9032). Reads were normalized against a calibration curve ranging from 0 to 250 μM MUF (*R*^2^ = 0.99). We used a conversion factor of 72 to convert the hydrolyzed conjugated fluorogenic substrate, measured as nM MUF to μg carbon (Hoppe, 1993).

### Statistical analysis

Data is displayed as average with error bars signifying one standard deviation (*n* = 7–9). All field relationships were determined with a Pearson correlation test (*n* = 7–9 and *P* < 0.01). Throughout all bottle incubations, significant differences between control and enriched microcosms were determined using Student's *t*-test with paired two-tailed distribution (*P* < 0.01). Changes in bacterial abundance were evaluated using One-Way analysis of variance (ANOVA) followed by Fisher LSD multiple comparison *post-hoc* test with a confidence of 95% using the XLSTAT software.

## Results and discussion

### Physicochemical properties of the Qishon estuary during the summer season

**Figure 2:**
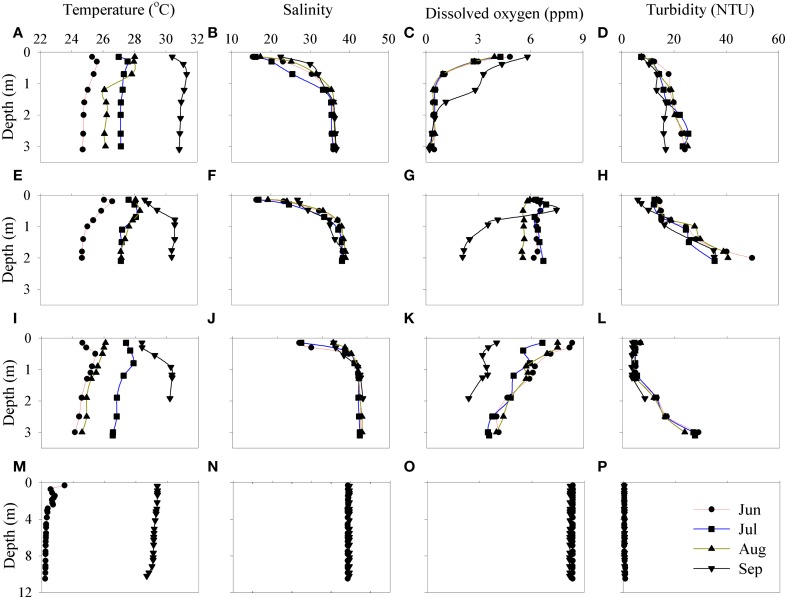
**Physicochemical depth profiles of the Qishon estuary stations downstream toward the coastal reference station; E3 (Histadrut)—(A–D), E1 (Maagan)—(E–H), E2 (Yulius)—(I–L), and Sh- (M–P)**. Measurements were taken between June and September.

The Qishon estuary is a 7 km long and shallow (~3 m deep) stream that flows through an industrial environment. During our study (June–September), no precipitation events were recorded (www.ims.gov.il), resulting in minimal freshwater supplements and slow flow rates (0.02–0.2 m^3^ s^−1^) (Vachtman et al., 2013). During the summer, daytime solar irradiance was high (~1500 μmol quanta m^−2^ s^−1^); yet, the turbid nature of this estuary system (Figure 2) minimized light penetration to surface layers (~0.2 m), limiting phytoplankton photo-damage (MacIntyre and Cullen, 1996). The surface of the estuary (upper 0.2 m) was saline (18–29), whereas the bottom (~3 m) was significantly saltier ranging from 36 to 39 (Figure 2, Table 1). The high salinity at the bottom was mainly due to eastern Mediterranean seawater percolating through from the sediment (Herut and Kress, 1997; Eliani-Russak et al., 2013). At all locations and sampling periods, temperature was mostly uniform (25–27°C) throughout the water column (Figure 2, Table 1). However, due to profound salinity differences, the estuary bottom layer was much denser than the surface (14 and 7.5 kg m^−3^ respectively), resulting in a strong stratification. The stable water column has possibly restricted vertical mixing and resulted in oxygen-depleted, low pH water at the bottom (Figure 2, Table 1) due to high microbial metabolic activities.

Effluents of two fertilizers plants, an oil refinery and a sewage treatment plant often flow into the Qishon estuary system, resulting in a eutrophic gradient (Table 1). Station E3, is located upstream and close to the above industrial effluents. As a result, dissolved inorganic nitrogen (NO_2_+ NO_3_, NH_4_, hereafter DIN) and phosphorus (DIP) concentrations were exceptionally high (543 ± 623 μM and 8.8 ± 4.5 μM respectively), with low DO levels (2.3 ± 2.2 mg L^−1^) indicating high bacterial activity (Table 1). Downstream, station E2 exhibited lower inorganic nutrient concentrations than E3, possibly due to gradual dilution, re-mineralization and sedimentation of aggregates (Table 1). Station E1 is located at the mouth of the estuary in a fishing harbor, and is therefore affected by small vessels, as well as mixing between the Qishon stream at the surface and coastal water at the bottom. Therefore, DIN was high at the surface (515 μM) and decreased by 7-fold near the bottom (72 μM). DO levels varied from 6.6 mg L^−1^ at the surface to 3.3 mg L^−1^ at the bottom and similar to E3 indicate high heterotrophic metabolism. Along the entire estuary, turbidity was significantly higher at the bottom (29 ± 11 NTU) than in the surface (8 ± 3 NTU) layer.

At the same time, a coastal station (Sh) uninfluenced by the estuary (Figure 1) was monitored as reference. The coastal station exhibited typical oligotrophic southeastern Mediterranean characteristics; low turbidity (~1.5 NTU), warm temperature (25 ± 3°C), high salinity (39 ± 0.4) and well oxidized water column (Figure 2, Table 1). DIN and DIP concentrations were 2–3 orders of magnitude lower than in the Qishon estuary (Table 1).

The eutrophic conditions in the Qishon water were probably due to anthropogenic effluents and the slow flow rate during the dry summer, resulting in a sharp nutrient gradient from the estuary toward the Mediterranean coast.

### Linking phytoplankton and bacterioplankton to TEP along the Qishon estuary system

**Figure 3:**
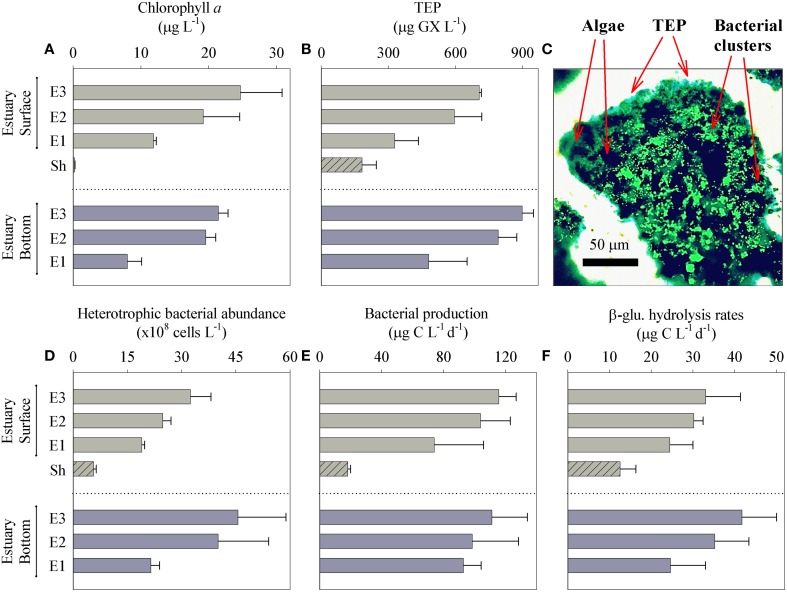
**Parameters sampled from the surface and bottom layers, along the eutrophic gradient; from Qishon estuary system (E3 to E1) to the coastal reference station (Sh)**. Water samples were analyzed for; Chlorophyll a as phytoplankton proxy **(A)**, TEP concentrations **(B)**, Microscopic visualization of a representative bio-aggregate **(C)**, heterotrophic bacterial abundance **(D)**, bacterial production **(E)**, and β-glucosidase activity **(F)**. Bars represent the average of three independent sampling dates with corresponding standard deviation. To visualize bio-aggregates water were sampled from a representative station (E1). Bacterial clusters were stained with SYTO 9 (bright green) and captured with epifluorescent microscopy. Bright field microscopy was used to visualize transparent exopolymers particles (TEP) stained with alcian blue (as light blue), and algal cells (as dark green). Images were superimposed using Image J software (http://rsbweb.nih.gov). Chlorophyll *a* pigment was not detected by autoflorescence within the bacterial clusters.

Phytoplankton biomass measured as Chl *a*, TEP concentrations, and heterotrophic bacterial abundance were all significantly higher in the Qishon estuary system compared with the reference coastal station (Figure 3). Chl *a* concentrations were 40–100-folds higher in the estuary than at the coastal station (Sh). A positive correlation was also found between phytoplankton concentration and distance from the coastal environment (*R*^2^ = 0.92, *P* < 0.001, *n* = 7), peaking at station E3; 24 ± 1 μg Chl *a* L^−1^ (Figure 3A and Figure S1A). We suggest that this significant relationship point on intense eutrophication, which resulted from sharp increase in nutrient availability (Table 1). However, phytoplankton was equally distributed along the depth profile of most measured stations, despite the turbid conditions in the Qishon estuary (Figure 3A, Table 1).

Along the Qishon estuary, TEP concentrations ranged between 225 and 1059 μg GX L^−1^ and were higher than the coastal station by 2–5-fold (Figure 3B). TEP were also found to increase linearly with the eutrophic gradient (*R*^2^ = 0.83, *P* < 0.001, *n* = 7), peaking at station E3 (Figure 3B and Figure S1B). However, TEP normalized to phytoplankton concentrations (μg GX Chl *a* ^−1^ L^−1^) followed an opposite trend and were up to 21-fold higher at the coastal station then the estuary. This opposing trend suggest that in the eutrophic Qishon estuary high TEP concentrations were the result of high phytoplankton biomass, while in the oligotrophic coastal environment induced physiological stress due to nutrient limitation may result in higher polysaccharide excretion per cell (Berman-Frank et al., 2007; Bar-Zeev et al., 2011, 2013). Moreover, it is possible that the high microplankton biomass within the estuary promoted viral infection that may result in cell lysis and TEP formation (Vardi et al., 2012; Lønborg et al., 2013).

TEP concentrations were consistently greater at the bottom in all estuary stations (Figure 3B). Sampled TEP were visualized as tightly packed, micrometer-size (hundreds of μm) bio-aggregates with numerous bacterial clusters held in a mixture of micro-algal cells (Figure 3C). No Chl *a* pigment was detected in the bacterial clusters based on epifluorescence microscopy, indicating that these microbial communities consisted primarily of heterotrophs (Figure 3C). These findings highlight the direct association between TEP mediated-aggregates and heterotrophic bacterial clusters. While drifting as aggregates, these newly formed, fresh-TEP are more labile (Passow, 2002) and are therefore likely to be utilized as substrates for bacterial growth. We suggest that the higher TEP concentrations at the bottom of the estuary were possibly due to polysaccharide release by benthic suspension feeders (McKee et al., 2005; Heinonen et al., 2007) and sedimentation processes, as previously shown for other estuaries and aquatic environments (Logan et al., 1995; Passow et al., 2001; Simon et al., 2002; Beauvais et al., 2006).

Heterotrophic bacterial abundances (BA) were 4–30-fold higher in the Qishon estuary (27 ± 14 × 10^8^ cells L^−1^) than at the coastal station (Figure 3D). Additionally, BA was found to gradually increase with distance from the coastal environment (*R*^2^= 0.75, *P* = 0.008, *n* = 7), peaking at the bottom of station E3 (Figure 3D and Figure S1C). Often BA comprises a mixture of bacteria with high nucleic acid (HNA) content and bacteria with low nucleic acid (LNA) content (Gasol and del Giorgio, 2000). HNA cells are usually larger and more active than LNA cells (Gasol and del Giorgio, 2000; Servais et al., 2003). In the Qishon estuary and the coastal station, HNA bacteria cells were 2–3-fold more abundant than LNA cells. These high ratios between HNA to LNA cells (>1) provide indirect indication that most of the bacterial community was highly active during the sampling period.

Along the Qishon estuary, bacterial production rates (BP) ranged from 74 to 121 μg C L^−1^ d^−1^, and were significantly higher (4–7-fold) than at the coastal station (Figure 3E). Similar to phytoplankton and BA, BP were also positively correlated with distance from the coastal environment (*R*^2^ = 0.83, *P* = 0.003, *n* = 7). All the above indicate that the microbial community was highly active throughout the estuary (Figure S1D). In fact, BP rates measured in the Qishon estuary were greater than most estuary systems (Ducklow and Carlson, 1992; Meon and Amon, 2004; Apple et al., 2006; Barrera-Alba et al., 2009; Santos et al., 2014), but were in agreement with rates reported from Pensacola Bay estuary in northwestern Florida (Murrell, 2003) and the Hudson River estuary (Findlay et al., 1991). Surprisingly, bacterial production per cell remained constant throughout the different sampling stations, ranging between 28 and 44 fg C cell^−1^ d^−1^. The sharp rise in BP and BA through the Qishon estuary, while retaining constant BP per cell, implies that the carbon assimilation rate reached a maximum of 36 ± 7 fg C cell^−1^ d^−1^ for the average bacterial cell.

β -glucosidase (β-glu) is one of various enzymes that are secreted by bacteria (hence defined as an ectoenzyme) to hydrolyze polysaccharides such as TEP into smaller, bio-available macromolecules (Radić et al., 2006; Engel et al., 2014). Similar to TEP, β-glu hydrolysis rates were significantly higher (2–3-fold) in all estuary samples than the coastal reference station and were positively correlated (*R*^2^ = 0.88, *P* = 0.002, *n* = 7) with distance from the coast (Figure 3F and Figure S1E). For all estuary sampling points, β-glu comprised 30% of the total assimilated carbon measured as BP, indicating on the importance of polysaccharide hydrolysis as a new-bioavailable carbon source in this system. Additionally, significant positive correlations were observed between TEP to bacterial abundance (*R*^2^ = 0.95, *P* < 0.001, *n* = 7) and β-glu activity (*R*^2^ = 0.93, *P* < 0.001, *n* = 7) (Figures S2A,B). We elucidate that this newly formed bioavailable carbon may than assimilated by bacteria according to cellular requirements. Further, the high hydrolysis rates by β-glu and the significant contribution to assimilated carbon (BP) indicate the importance of polysaccharides to bacterial metabolism in the Qishon estuary.

From the field observations, we conclude that high BA and TEP concentrations in the Qishon estuary increased the probability of polysaccharide-hydrogels to be colonized by bacteria and form bio-aggregates. The significant contribution of β-glu to BP and the positive correlations with TEP are all indirect indications that these polysaccharides were hydrolyzed to form an additional carbon source for heterotrophic bacterial proliferation.

### TEP contribution to bacterial activity in confined bottle incubations

The direct link between TEP and heterotrophic bacterial metabolism was further characterized by carrying out 18 bottle-microcosms. To model TEP contribution to bacterial activity, gum xanthan (GX) was added to nine “enriched” bioassays as pure polysaccharide substrate, since the chemical composition of naturally formed TEP varies drastically (Passow, 2002). Changes in BA, heterotrophic bacterial composition (LNA and HNA), metabolic activity, TEP concentrations and ectoenzymatic hydrolysis rates were measured at the beginning and end of the incubation. Values from the control (un-amended estuary water) incubations were than compared to the enriched (GX) microcosms.

Water samples (1L) were collected from the surface and the bottom of stations E1–E3 along the Qishon estuary, during the summer months (July and August, 2014). To focus on the bacterial community, sampled water were pre-filtered (1 μm) to remove any large suspended particles and micro-phytoplankton. As a result, most phytoplankton were dismissed, evident by the drastic reduction (~90%) in Chl *a* concentrations, while most heterotrophic bacterioplankton were retained (70–90%). Additionally, it is likely that most grazers such as copepods (e.g., *Acartia* sp.) and isopods (e.g., *Cirolana* sp.) were also removed by the pre-filtration step. To prioritize bacterial heterotrophs while maintaining the native physicochemical conditions of our study site, all microcosm bottles were incubated under dark conditions for 2 days (T_2d_).

#### Un-amended, control bioassays

**Figure 4:**
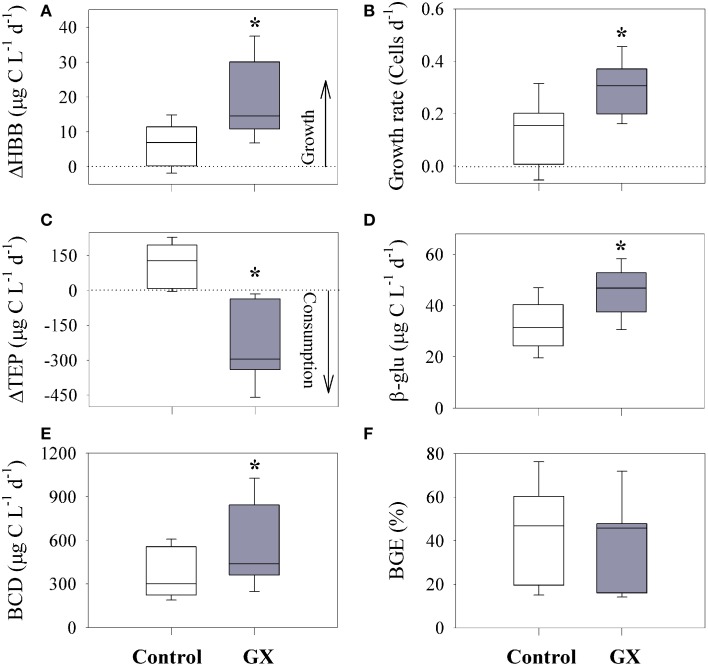
**Differences between the control and GX-enriched microcosm experiments comparing: changes in heterotrophic bacterial biomass (ΔHBB = T_2_-T_0_) (A), growth rates (B) and TEP concentrations (ΔTEP = T_2_-T_0_) (C) as well as β-glucosidase activity (β-glu) (D), bacterial carbon demand (BCD) (E), and bacterial growth efficiency (BGE) (F)**. Each box plot represents microcosms that were incubated with water samples from the surface and bottom of stations E1–E3. Box plot boundary are the 25th–75th percentile while the inner line represent median and whiskers highlight the 10th–90th percentile. Significant differences between control and GX enriched microcosms are marked with an asterisk (*P* < 0.01).

**Figure 5:**
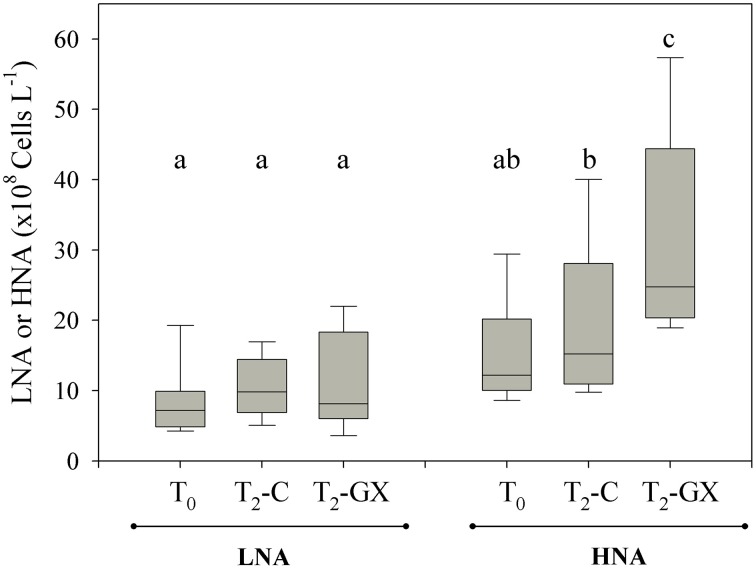
**Changes in heterotrophic bacterial community composition following GX addition (GX) compared to un-amended controls (C) during the experimental microcosm 2 days incubations (T_0_–T_2_)**. The bacterial community was segregated into two subgroups containing high and low nucleic acid content (HNA and LNA, respectively). Each box plot represents microcosms that were carried with water samples from the surface and bottom of stations E1–E3. Differences between HNA and LNA were evaluated using One-Way ANOVA followed by Fisher LSD multiple comparison *post-hoc* test with a confidence of 95%.

Over the course of the incubations (T_0_–T_2d_), phytoplankton decreased by 83 ± 17%, while BA and corresponding heterotrophic bacterial biomass (HBB) increased by 32 ± 31% (Figure 4A, Table 2). During this time, the heterotrophic bacterial community showed no change in the HNA/LNA ratio (~2) compared to the initial (T_0_) conditions (Figure 5, Table 2). Although BA was high at the end of the control incubations in respect to initial concentrations, growth rates were exceptionally low (Figure 4B, Table 2) compared to other marine and fresh water environments (Hamasaki et al., 2004; Smith and Benner, 2005; Azam and Malfatti, 2007; Barrera-Alba et al., 2009).

**Table 2:** **Biological properties measured in un-amended controls and gum-xanthan (GX) enriched microcosms incubated 2 days (T_2d_) under dark + DCMU conditions**.

**Parameter**	**Units**	**Control**		**GX enriched**	
		**T_0_**	**T_2d_**	**T_0_**	**T_2d_**
Chl. *a*	μg L^−1^	2.1±0.6	0.3±0.2	2.1±0.6	0.3±0.3
HBB^a^	μg C L^−1^	47±22	60±29	47±22	86±44
HNA	x10^8^ Cell L^−1^	15±7.1	20±10	15±7.1	31±14
LNA	x10^8^ Cell L^−1^	8.3±4.6	10±4.2	8.3±4.6	11±6.3
HNA/LNA	Cell/Cell	2±0.7	1.9±0.6	2±0.7	3.3±1.3
TEP^b^	μg C L^−1^	160±48	384±206	1312±52	849±351
β-glu^c^	μg C L^−1^ d^−1^	34±7.5	32±9	34±7.5	46±9
BP^d^	μg C L^−1^ d^−1^	109±21	165±34	109±21	121±24
BCD	μg C L^−1^ d^−1^	NA	359±167	NA	557±276
BGE	%	NA	42±22	NA	37±20
Growth rate^e^	d^−1^	NA	0.15±0.1	NA	0.28±0.09

*All values are the averages and their standard deviation of nine bottle incubations*.

a *Bacterial abundance was converted into biomass (HBB) by a factor of 20 fg C per cell (Lee and Fuhrman, 1987)*.

b *TEP was converted from GX into carbon biomass using a 0.74 factor (Engel and Passow, 2001)*.

c *β-glucosidase activity (β-glu) was converted from MUF to carbon biomass using 72 μg C to 1 μM MUF factor (Hoppe, 1993)*.

d *Bacterial production was converted into carbon biomass using a conversion factor of 3.1 kg C mol^−1^ with an isotope dilution factor of 2.0 to calculate BP (Simon and Azam, 1989)*.

e *Growth rates calculated according to Equation (1)*.

**Figure 6:**
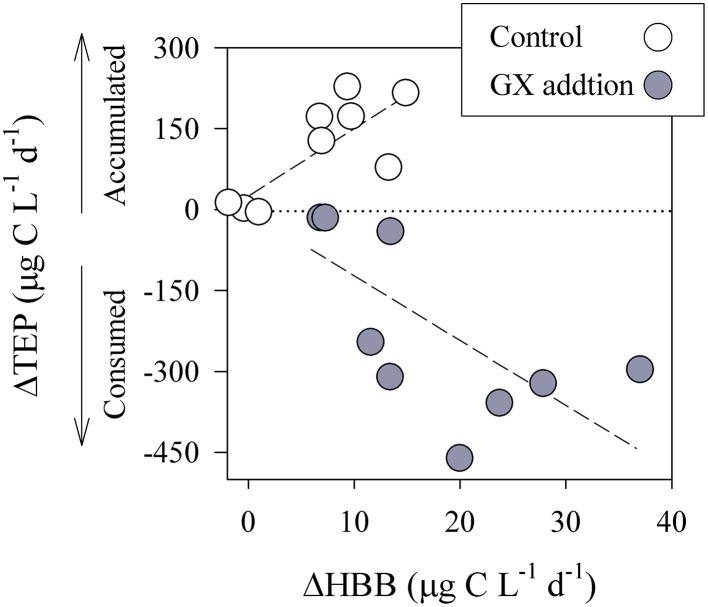
**Relationship between changes in TEP concentrations (ΔTEP = T_2_-T_0_) and differences in heterotrophic bacterial biomass (ΔHBB = T_2_-T_0_)**. Data was collected from both the control (white circles) and GX enriched (gray circles) bottle incubations. Linear regression is shown of: ΔTEP vs. ΔHBB for the control (*R*^2^ = 0.54, *P* = 0.014, *n* = 9) and enriched (*R*^2^ = 0.40, *P* = 0.09, *n* = 9) microcosms.

Concurrently, initial TEP concentrations increased by up to 2.4 ± 1-fold resulting in polysaccharide accumulation, ΔTEP; T_2d_-T_0_ > 0 (Figure 4C, Table 2). Further, TEP concentrations were found to positively correlate with the increase in heterotrophic bacterial biomass (Figure 6). The accumulation of TEP, the positive correlation with heterotrophic bacterial growth (ΔHBB; T_2d_-T_0_ > 0) and the extremely low phytoplankton concentrations, all indicate that in the control incubations TEP was mostly generated by the heterotrophic bacterial community. We suggest that some of the polysaccharide secretions were triggered by viruses that passed filtration and may infect the bacterial community as previously suggested for phytoplankton (Vardi et al., 2012; Lønborg et al., 2013). Concurrent to these direct bacterial TEP secretions, TEP precursors (5–400 nm) that passed the filtration step may self-assembled during the incubation by diffusion and electrostatic interaction to form larger, new TEP (Chin et al., 1998; Verdugo, 2012; Bar-Zeev et al., 2015).

Polysaccharide hydrolysis by β-glucosidase activity (β-glu) ranged between 20 and 47 μg C L^−1^ d^−1^ (Figure 4D), using a conversion factor of 72 μg C to 1 μM MUF (Hoppe, 1993). These ectoenzyme activity rates were relatively high compared to those observed in other eutrophic and oligotrophic environments (Rath et al., 1993; Martinez et al., 1996; Radić et al., 2006). However, they were lower than reported around oil slicks as high hydrocarbon source (Ziervogel et al., 2014). Cell specific hydrolysis rates (12 ± 5 fg C cell^−1^ d^−1^) were also higher than values reported from the open ocean (Baltar et al., 2009), but highly comparable to hydrolysis rates by bacteria that harbored aggregates (Karner and Herndl, 1992; Azúa et al., 2003; Corinaldesi et al., 2003).

Bacterial respiration (BR) and bacterial production (BP) have varied between the nine incubations, ranging from 45 to 489 μg C L^−1^ d^−1^ and 86 to 162 μg C L^−1^ d^−1^, respectively. Carbon oxidation through BR (measured as oxygen reduction) was determined following two key assumptions: (i) since most phytoplankton were removed through size exclusion and hindered by the darken incubations, we estimated that community respiration was ascribed mostly (~ 90%) to microbial heterotrophs. (ii) Converting oxygen consumption to carbon utilization was done by a conservative respiratory-quotient (RQ) of 1 (del Giorgio and Cole, 1998; Anesio et al., 2003; Smith and Prairie, 2004).

Heterotrophic metabolism rates were derived from both anabolic (BP) and catabolic (BR) reactions, hereafter defined as bacterial carbon demand (BCD = BP+BR) and bacterial growth efficiency (BGE = BP/BCD). In the control incubations, calculated BCD was 359 ± 167 μg C L^−1^ d^−1^ and BGE values were 42 ± 22%, *n* = 9 (Figures 4E,F). BCD corresponds to the carbon biomass that is required to sustain the net bacterial metabolic needs, while BGE values point on the efficiency at which carbon is assimilated into bacterial biomass (del Giorgio and Cole, 1998; Ducklow et al., 2000). Since BP was determined by short (4 h) incubations, while BR was estimated by measuring oxygen consumption over 48 h, BCD and BGE may have been overestimated to some extent. Further bias might result from variations in bacterial community composition (e.g., HNA: LNA) and by using a conservative RQ of 1, whereas values are known to vary between 0.5 and 4, depending on the oxidation potential of organic compounds (Berggren et al., 2012). Nevertheless, both BCD and BGE measured in this study (neglecting two, high outgroups) were highly comparable with other eutrophic, marine and fresh water environments (Apple and del Giorgio, 2007; Bhaskar and Bhosle, 2008; Amado et al., 2013; Ziervogel et al., 2014).

We suggest that although heterotrophic bacterial abundance (BA) was higher at the end of the control incubations than initial values, the low growth rates relative to other environments indicate that metabolic activity was mostly tunneled to maintain cellular energetic requirements rather than growth and propagation. These results imply that even in a eutrophic environment such as the Qishon estuary, bacterial heterotrophs were possibly nutrient (e.g., carbon) deficient.

#### Gum xanthan (GX) enriched bioassays

Following GX additions (comprising glucose, mannose and glucuronic acid), TEP concentrations increased by 8-fold compared to the control (un-amended) microcosms (Table 2). By the end of the incubations (T_0_–T_2d_), heterotrophic bacterial growth rates doubled compared to the control, resulting in 80 ± 40% increase in BA (Figures 4A,B, Table 2). The raise in BA was mostly attributed to drastic HNA proliferation (Figure 5, Table 2). HNA bacterial activity was shown in both laboratory experiments (Servais et al., 2003) and elevated hydrolysis of carbohydrates in field studies (Piontek et al., 2014) highlighting the potential of HNA to proliferate in carbon enriched systems.

Compared to the control incubations, ΔHBB (T_2d_- T_0_) significantly increased following GX enrichment, while TEP was reduced (ΔTEP < 0), pointing on intense polysaccharide hydrolysis (Figures 4A,C). Moreover, the negative correlation found between ΔTEP and ΔHBB (Figure 6) is an additional indication that heterotrophic bacterial growth was linked to TEP consumption.

We suggest that following the addition of GX, TEP concentrations decreased through enhanced ectoenzymatic (β-glu) activity relative to the initial rates, T_2_ > T_0_; 36 ± 17% (Table 2). Further, at the end of the enriched incubations, β-glu activity was significantly higher than measured at the control bottles (Figure 4D). These intense hydrolysis rates by β-glu were likely triggered by the polysaccharide (GX) substrates, as well as the close proximity of the ectoenzymes released by bacteria harboring TEP (Simon et al., 2002; Azam and Malfatti, 2007; Stocker, 2012). We determined that this newly formed carbon comprised 51 ± 43% of the heterotrophic BCD in the nine enriched bioassays. This bioavailable source of carbon could then prompt bacterial growth by enhancing both anabolic and catabolic reactions (Figure 4E). High BCD, through positive feedback-loops may result in constant polysaccharide release, forming TEP that could then be hydrolyzed as fresh, available carbon source.

In contrast to BCD, no changes in average BGE (using RQ of 1) were measured following GX addition compared to the control incubations (Figure 4F), since both BP and BR increased in a similar ratio (40 and 50% respectively). We do acknowledge that variations in the organic composition may shift RQ and alter BGE. However, adjusting RQ to 1.2 as previously suggested for glucose (Berggren et al., 2012) did not significantly change BGE values.

From all the confined incubations we deduced that GX addition as newly formed polysaccharide-rich substrates were most likely harbored by bacteria forming “hotspots” with enhanced BCD. We suggest that the high metabolic requirements were paired with intense ectoenzymatic (e.g., β-glu) activity, hydrolyzing polysaccharide chains into bioavailable organic compounds (Hoppe, 1993; Martinez et al., 1996; Simon et al., 2002; Piontek et al., 2014). The consequence of that newly introduced carbon source, even in a highly eutrophic environment such as the Qishon estuary was heterotrophic microbial proliferation. Yet, it is possible that once carbon is available, other nutrient might become limiting, thereby specialized heterotrophic bacteria such as diazotrophs could utilize these new conditions (e.g., high C > N) and prevail (Rahav et al., 2013).

## Conclusion

Our study, carried out at the field and through bottle-bioassays, was designed to link TEP to heterotrophic metabolism and bacterial growth in a eutrophic estuary system. Along the Qishon estuary TEP concentrations gradually increased, concurrently with the development of phytoplankton and bacterioplankton biomass, most probably through active excretion of polymeric substances (Figure 3). TEP were often found as large bio-aggregates, scaffolding algae, bacteria and detritus matter (Figure 3C). The tight association between TEP, as organic substrate, and bacteria has facilitated polysaccharide hydrolysis through intense β-glu-ectoenzymatic activity (Figures 3, 4).

We postulate that this newly bioavailable carbon source is formed and consumed along the Qishon estuary through a positive feedback loop: (i) initially, bacterial anabolic and catabolic reactions are prompt, thereby increasing heterotrophic growth rates, mainly of the HNA subgroup (Figure 5). (ii) While proliferating, bacteria constantly secrete dissolved and particulate organic matter that forms new TEP, which may later be hydrolyzed.

Based upon the results above we deduce that heterotrophic bacteria were limited by carbon, despite the eutrophic nature of the Qishon estuary. Hence, regardless of the carbon source (autotrophic, heterotrophic or anthropogenic), when fresh TEP (e.g., GX) are introduced to the Qishon eutrophic-water, the bacterial community proliferates. Currently, we cannot rule-out co-limitation by other microelements such as phosphorous and/or nitrogen once carbon is supplemented. Regardless, it is clear that in the Qishon estuary and likely in similar systems, TEP act as a dynamic metabolic link fueling the microbial loop, concurrently to other possible roles such as aggregation, sedimentation and pathogen transfer.

## Author contributions

EB and ER designed the research; ER conducted the experiments; EB and ER analyzed the data; EB and ER wrote the paper.

### Conflict of interest statement

The authors declare that the research was conducted in the absence of any commercial or financial relationships that could be construed as a potential conflict of interest.

## References

[B1] Alldredge A. L. Passow U. Logan B. E. (1993). The abundance and significance of a class of large, transparent organic particles in the ocean. Deep Sea Res. Part I 40, 1131–1140. 10.1016/0967-0637(93)90129-Q

[B2] Amado A. M. Meirelles-Pereira F. Vidal L. O. Sarmento H. Suhett A. L. Farjalla V. F.. (2013). Tropical freshwater ecosystems have lower bacterial growth efficiency than temperate ones. Front. Microbiol. 4:167. 10.3389/fmicb.2013.00167 23801986 PMC3689033

[B3] Anesio A. M. Abreu P. C. Biddanda B. A. (2003). The role of free and attached microorganisms in the decomposition of estuarine macrophyte detritus. Estuar. Coast. Shelf Sci. 56, 197–201. 10.1016/S0272-7714(02)00152-X

[B4] Apple J. K. del Giorgio P. A. (2007). Organic substrate quality as the link between bacterioplankton carbon demand and growth efficiency in a temperate salt-marsh estuary. ISME J. 1, 729–742. 10.1038/ismej.2007.86 18059496

[B5] Apple J. K. Del Giorgio P. A. Kemp W. M. (2006). Temperature regulation of bacterial production, respiration, and growth efficiency in a temperate salt-marsh estuary. Aquat. Microb. Ecol. 43, 243–254. 10.3354/ame043243

[B6] Azam F. (1998). Microbial control of oceanic carbon flux: the plot thickens. Science 280, 694–696. 10.1126/science.280.5364.694

[B7] Azam F. Malfatti F. (2007). Microbial structuring of marine ecosystems. Nat. Rev. Microbiol. 5, 782–791. 10.1038/nrmicro1747 17853906

[B8] Azúa I. Unanue M. Ayo B. Artolozaga I. Arrieta J. M. Iriberri J. (2003). Influence of organic matter quality in the cleavage of polymers by marine bacterial communities. J. Plankton Res. 25, 1451–1460. 10.1093/plankt/fbg105

[B9] Baltar F. Arístegui J. Sintes E. Van Aken H. M. Gasol J. M. Herndl G. J. (2009). Prokaryotic extracellular enzymatic activity in relation to biomass production and respiration in the meso- and bathypelagic waters of the (sub)tropical Atlantic. Environ. Microbiol. 11, 1998–2014. 10.1111/j.1462-2920.2009.01922.x 19508555

[B10] Barrera-Alba J. J. Gianesella S. M. F. Moser G. A. O. Saldanha-Corrêa F. M. P. (2009). Influence of allochthonous organic matter on bacterioplankton biomass and activity in a eutrophic, sub-tropical estuary. Estuar. Coast. Shelf Sci. 82, 84–94. 10.1016/j.ecss.2008.12.020

[B11] Bar-Zeev E. Avishay I. Bidle K. D. Berman-Frank I. (2013). Programmed cell death in the marine cyanobacterium *Trichodesmium* mediates carbon and nitrogen export. ISME J. 7, 2340–2348. 10.1038/ismej.2013.121 23887173 PMC3834853

[B12] Bar-Zeev E. Berman T. Rahav E. Dishon G. Herut B. Berman-Frank I. (2011). Transparent exopolymer particle (TEP) dynamics in the eastern Mediterranean Sea. Mar. Ecol. Prog. Ser. 431, 107–118. 10.3354/meps09110

[B13] Bar-Zeev E. Passow U. Romero-Vargas Castrillón S. Elimelech M. (2015). Transparent exopolymer particles: from aquatic environments and engineered systems to membrane biofouling. Environ. Sci. Technol. 49, 691–707. 10.1021/es5041738 25494664

[B14] Beauvais S. Pedrotti M. Egge J. Iversen K. Marrasé C. (2006). Effects of turbulence on TEP dynamics under contrasting nutrient conditions: implications for aggregation and sedimentation processes. Mar. Ecol. Prog. Ser. 323, 47–57. 10.3354/meps323047

[B15] Berggren M. Lapierre J.-F. del Giorgio P. A. (2012). Magnitude and regulation of bacterioplankton respiratory quotient across freshwater environmental gradients. ISME J. 6, 984–993. 10.1038/ismej.2011.157 22094347 PMC3329109

[B16] Berman T. Viner-mozzini Y. (2001). Abundance and characteristics of polysaccharide and proteinaceous particles in Lake Kinneret. Aquat. Microb. Ecol. 24, 255–264. 10.3354/ame024255

[B17] Berman-Frank I. Rosenberg G. Levitan O. Haramaty L. Mari X. (2007). Coupling between autocatalytic cell death and transparent exopolymeric particle production in the marine cyanobacterium *Trichodesmium*. Environ. Microbiol. 9, 1415–1422. 10.1111/j.1462-2920.2007.01257.x 17504479

[B18] Bhaskar P. V. Bhosle N. B. (2008). Bacterial production, glucosidase activity and particle-associated carbohydrates in Dona Paula bay, west coast of India. Estuar. Coast. Shelf Sci. 80, 413–424. 10.1016/j.ecss.2008.09.005

[B19] Carpenter J. H. (1965). The Chesapeake Bay Institute technique for the Winkler dissolved oxygen method. Limnol. Oceanogr. 10, 141–143. 10.4319/lo.1965.10.1.0141

[B20] Chin W.-C. Orellana M. V. Verdugo P. (1998). Spontaneous assembly of marine dissolved organic matter into polymer gels. Lett. Nat. 391, 568–572. 10.1038/35345

[B21] Corinaldesi C. Crevatin E. Negro P. Del Marini M. Danovaro R. Russo A. (2003). Large-scale spatial distribution of virioplankton in the Adriatic Sea: testing the trophic state control hypothesis. Appl. Environ. Microbiol. 69, 2664–2673. 10.1128/AEM.69.5.2664-2673.2003 12732535 PMC154510

[B22] Decho A. W. Lopez G. R. (1993). Exopolymer microenvironments of microbial flora: multiple and interactive effects on trophic relationships. Limnol. Oceanogr. 38, 1633–1645. 10.4319/lo.1993.38.8.1633

[B23] de La Rocha C. L. Passow U. (2007). Factors influencing the sinking of POC and the efficiency of the biological carbon pump. Deep Sea Res. Part II Top. Stud. Oceanogr. 54, 639–658. 10.1016/j.dsr2.2007.01.004

[B24] del Giorgio P. A. Cole J. J. (1998). Bacterial growth efficiency in natural aquatic systems. Annu. Rev. Ecol. Syst. 29, 503–541. 10.1146/annurev.ecolsys.29.1.503

[B25] Ducklow H. W. Carlson C. A. (1992). Oceanic bacterial production, in Advances in Microbial Ecology, ed Marshall K. C. (New York, NY: Plenum Press), 113–181.

[B26] Ducklow H. W. Dickson M. Kirchman D. L. Steward G. Orchardo J. Marra J. (2000). Constraining bacterial production, conversion efficiency and respiration in the Ross Sea. Deep. Res. II. 47, 3227–3247. 10.1016/S0967-0645(00)00066-7

[B27] Eliani-Russak E. Herut B. Sivan O. (2013). The role of highly sratified nutrient-rich small estuaries as a source of dissolved inorganic nitrogen to coastal seawater, the Qishon (SE Mediterranean) case. Mar. Pollut. Bull. 71, 250–258. 10.1016/j.marpolbul.2013.02.001 23485104

[B28] Elliott M. McLusky D. S. (2002). The need for definitions in understanding estuaries. Estuar. Coast. Shelf Sci. 55, 815–827. 10.1006/ecss.2002.1031

[B29] Engel A. (2004). Distribution of transparent exopolymer particles (TEP) in the northeast Atlantic Ocean and their potential significance for aggregation processes. Deep Sea Res. Part I 51, 83–92. 10.1016/j.dsr.2003.09.001

[B30] Engel A. Passow U. (2001). Carbon and nitrogen content of transparent exopolymer particles (TEP) in relation to their alcian blue adsorption. Mar. Ecol. Prog. Ser. 219, 1–10. 10.3354/meps219001

[B31] Engel A. Piontek J. Grossart H. Riebesell U. L. F. Schulz K. A. I. G. Sperling M. (2014). Impact of CO_2_ enrichment on organic matter dynamics during nutrient induced coastal phytoplankton blooms. J. Plankt. Res. 36, 641–657. 10.1093/plankt/fbt125

[B32] Findlay S. Pace M. L. Lints D. Cole J. J. Caraco N. F. Peierls B. (1991). Weak coupling of bacterial and algal production in a heterotrophic ecosystem: the Hudson River estuary. Limnol. Oceanogr. 36, 268–278. 10.4319/lo.1991.36.2.0268

[B33] Gasol J. M. del Giorgio P. A. (2000). Using flow cytometry for counting natural planktonic bacteria and understanding the structure of planktonic bacterial communities. Sci. Mar. 64, 197–224. 10.3989/scimar.2000.64n2197

[B34] Grossart H. Simon M. Logan B. E. (1997). Frormation or macroscopic organic aggregates (lake snow) in a large lake: the significance of transparent exopolymer particles, phytoplankton, and zooplankton. Limnol. Oceanogr. 42, 1651–1659. 10.4319/lo.1997.42.8.1651

[B35] Grossart H.-P. Czub G. Simon M. (2006). Algae-bacteria interactions and their effects on aggregation and organic matter flux in the sea. Environ. Microbiol. 8, 1074–1084. 10.1111/j.1462-2920.2006.00999.x 16689728

[B36] Hall N. Pearl H. (2011). Vertical migration patterns of phytoflagellates in relation to light and nutrient availability in a shallow microtidal estuary. Mar. Ecol. Prog. Ser. 425, 1–19. 10.3354/meps09031

[B37] Hamasaki K. Long R. A. Azam F. (2004). Individual cell growth rates of marine bacteria, measured by bromodeoxyuridine incorporation. Aquat. Microb. Ecol. 35, 217–227. 10.3354/ame035217

[B38] Heinonen K. B. Ward J. E. Holohan B. A. (2007). Production of transparent exopolymer particles (TEP) by benthic suspension feeders in coastal systems. J. Exp. Mar. Bio. Ecol. 341, 184–195. 10.1016/j.jembe.2006.09.019

[B39] Herut B. Kress N. (1997). Particulate metals contamination in the Kishon River estuary, Israel. Mar. Pollut. Bull. 34, 706–711. 10.1016/S0025-326X(97)00018-0

[B40] Herut B. Tibor G. Yacobi Y. Z. Kress N. Image N. Sensing R. (1999). Synoptic measurements of Chlorophyll *a* and suspended particulate matter in a transitional zone from polluted to clean seawater utilizing airborne remote sensing and ground measurements, Haifa Bay (SE Mediterranean). Mar. Pollut. Bull. 38, 762–772. 10.1016/S0025-326X(99)00038-7

[B41] Holm-Hansen O. Lorenzen C. J. Holmes R. W. Strickland J. D. (1965). Fluorometric determination of chlorophyll. J. Cons. 30, 3–15. 10.1093/icesjms/30.1.3

[B42] Hoppe H.-G. (1993). Use of fluorogenic model substrates for extracellular enzyme activity (EEA) measurement of bacteria, in Handbook of Methods in Aquatic Microbial Ecology, eds Kemp P. F. Sherr B. F. Sherr E. B. Cole J. J. (Boca Raton, FL: Lewis Publishers), 423–431.

[B43] Karner M. Herndl G. J. (1992). Extracellular enzymatic activity and secondary production in free-living and marine snow associated bacteria. Mar. Biol. 113, 341–347.

[B44] Kress N. Gertman I. Herut B. (2014). Temporal evolution of physical and chemical characteristics of the water column in the Easternmost Levantine basin (Eastern Mediterranean Sea) from 2002 to 2010. J. Mar. Syst. 135, 6–13. 10.1016/j.jmarsys.2013.11.016

[B45] Kress N. Herut B. (2001). Spatial and seasonal evolution of dissolved oxygen and nutrients in the southern Levantine Basin (Eastern Mediterranean Sea): chemical characterization of the water masses and inferences on the N: P ratios. Deep. Res. I 48, 2347–2372. 10.1016/S0967-0637(01)00022-X

[B46] Krom M. D. Brenner S. Israilov L. Krumgalz B. (1991). Dissolved nutrients, preformed nutrients and calculated elemental ratios in the South-East Mediterranean Sea. Oceanol. Acta 14, 189–194.

[B47] Lee S. Fuhrman J. A. (1987). Relationships between biovolume and biomass of naturally derived marine bacterioplankton. Appl. Environ. Microbiol. 53, 1298–1303. 16347362 10.1128/aem.53.6.1298-1303.1987 PMC203858

[B48] Logan B. E. Passow U. Alldredge A. L. Grossartt H.-P. Simon M. (1995). Rapid formation and sedimentation of large aggregates is predictable from coagulation rates (half-lives) of transparent exopolymer particles (TEP). Deep Sea Res. Part II 42, 203–214. 10.1016/0967-0645(95)00012-F

[B49] Lønborg C. Middelboe M. Brussaard C. P. (2013). Viral lysis of *Micromonas pusilla*: impacts on dissolved organic matter production and composition. Biogeochemistry 116 231–240. 10.1007/s10533-013-9853-1

[B50] Luna G. M. Bianchelli S. Decembrini F. De Domenico E. Danovaro R. Dell'Anno A. (2012). The dark portion of the Mediterranean Sea is a bioreactor of organic matter cycling. Global Biogeochem. Cycles 26, GB2017 10.1029/2011GB004168

[B51] Lyons M. M. Lau Y.-T. Carden W. E. Ward J. E. Roberts S. B. Smolowitz R. (2007). Characteristics of marine aggregates in shallow-water ecosystems: implications for disease ecology. Ecohealth 4, 406–420. 10.1007/s10393-007-0134-0

[B52] MacIntyre H. L. Cullen J. J. (1996). Primary production by suspended and benthic microalgae in a turbid estuary: time-scales of variability in San Antonio Bay, Texas. Mar. Ecol. Prog. Ser. 145, 245–268. 10.3354/meps145245

[B53] Malone T. C. Conley D. J. Fistter T. R. Sellner K. G. (1996). Scales of nutrient-limited phytoplankton productivity in Chesapeake Bay. Estuaries 19, 371–385. 10.2307/1352457

[B54] Mari X. Kirboe T. (1996). Abundance, size distribution and bacterial colonization of transparent exopolymeric particles (TEP) during spring in the Kattegat. J. Plankton Res. 18, 969–986. 10.1093/plankt/18.6.969

[B55] Mari X. Torréton J.-P. Bich-Thuy Trinh C. Bouvier T. Van Thuoc C. Lefebvre J.-P. (2012). Aggregation dynamics along a salinity gradient in the Bach Dang estuary, North Vietnam. Estuar. Coast. Shelf Sci. 96, 151–158. 10.1016/j.ecss.2011.10.028

[B56] Martinez J. Smith C. Steward G. F. Azam F. (1996). Glucosidase activity. Aquat. Microb. Ecol. 10, 223–230. 10.3354/ame010223

[B57] McKee M. P. Ward J. E. MacDonald B. A. Holohan B. A. (2005). Production of transparent exopolymer particles (TEP) by the eastern oyster *Crassostrea virginica*. Mar. Ecol. Prog. Ser. 288, 141–149. 10.3354/meps288141

[B58] Meon B. Amon R. M. (2004). Heterotrophic bacterial activity and fluxes of dissolved free amino acids and glucose in the Arctic rivers Ob, Yenisei and the adjacent Kara Sea. Aquat. Microb. Ecol. 37, 121–135. 10.3354/ame037121

[B59] Murrell M. C. (2003). Bacterioplankton dynamics in a subtropical estuary: evidence for substrate limitation. Aquat. Microb. Ecol. 32, 239–250. 10.3354/ame032239

[B60] Passow U. (2002). Transparent exopolymer particles (TEP) in aquatic environments. Prog. Oceanogr. 55, 287–333. 10.1016/S0079-6611(02)00138-6

[B61] Passow U. Alldredge A. L. (1995). A dye-binding assay for the spectrophotometric measurement of transparent exopolymer particles (TEP). Limnol. Oceanogr. 40, 1326–1335. 10.4319/lo.1995.40.7.1326

[B62] Passow U. Shipe R. F. Murray A. Pak D. K. Brzezinski M. A. Alldredge A. (2001). The origin of transparent exopolymer particles (TEP) and their role in the sedimentation of particulate matter. Cont. Shelf Res. 21, 327–346. 10.1016/S0278-4343(00)00101-1

[B63] Pinckney J. L. Paerl H. W. Tester P. Richardson T. L. (2001). The role of nutrient loading and eutrophication in Estuarine ecology a definition of eutrophication. Environ. Heal. Prespect. 109, 699–706. 10.1289/ehp.01109s5699 11677178 PMC1240600

[B64] Piontek J. Sperling M. Nöthig E.-M. Engel A. (2014). Regulation of bacterioplankton activity in Fram Strait (Arctic Ocean) during early summer: the role of organic matter supply and temperature. J. Mar. Syst. 132, 83–94. 10.1016/j.jmarsys.2014.01.003

[B65] Pritchard D. W. (1967). What is an estuary: physical viewpoint. Estuaries 83, 3–5. 11760185

[B66] Radić T. Ivancić I. Fuks D. Radić J. (2006). Marine bacterioplankton production of polysaccharidic and proteinaceous particles under different nutrient regimes. FEMS Microbiol. Ecol. 58, 333–342. 10.1111/j.1574-6941.2006.00176.x 17117978

[B67] Rahav E. Bar-Zeev E. Ohayon S. Elifantz H. Belkin N. Herut B.. (2013). Dinitrogen fixation in aphotic oxygenated marine environments. Front. Microbiol. 4:227. 10.3389/fmicb.2013.00227 23986748 PMC3753716

[B68] Rath J. Schiller C. Herndl G. J. (1993). Ectoenzymatic activity and bacterial dynamics along a trophic gradient in the Caribbean Sea. Mar. Ecol. Prog. Ser. 102, 89–96. 10.3354/meps102089

[B70] Santos L. Vaz L. Gomes N. C. M. Vaz N. Dias J. M. Cunha Â. (2014). Impact of freshwater inflow on bacterial abundance and activity in the estuarine system Ria de Aveiro. Estuar. Coast. Shelf Sci. 138, 107–120. 10.1016/j.ecss.2013.12.021

[B71] Servais P. Casamayor E. O. Courties C. Catala P. Parthuisot N. Lebaron P. (2003). Activity and diversity of bacterial cells with high and low nucleic acid content. Aquat. Microb. Ecol. 33, 41–51. 10.3354/ame033041

[B72] Simon M. Alldredge A. L. Azam F. (1990). Bacterial carbon dynamics on marine snow. Mar. Ecol. Prog. Ser. 65, 205–211. 10.3354/meps065205

[B73] Simon M. Azam F. (1989). Protein content and protein synthesis rates of planktonic marine bacteria. Mar. Ecol. Prog. Ser. 51, 201–213. 10.3354/meps051201

[B74] Simon M. Grossart H. Schweitzer B. Ploug H. (2002). Microbial ecology of organic aggregates in aquatic ecosystems. Aquat. Microb. Ecol. 28, 175–211. 10.3354/ame02817523250114

[B75] Smith D. C. Azam F. (1992). A simple, economical method for measuring bacterial protein synthesis rates in seawater using ^3^H-leucine. Mar. Microb. Food Webs 6, 107–114.

[B76] Smith E. M. Benner R. (2005). Photochemical transformations of riverine dissolved organic matter: effects on estuarine bacterial metabolism and nutrient demand. Aquat. Microb. Ecol. 40, 37–50. 10.3354/ame040037

[B77] Smith E. M. Prairie Y. T. (2004). Bacterial metabolism and growth efficiency in lakes: the importance of phosphorus availability. Limnol. Oceanogr. 49, 137–147. 10.4319/lo.2004.49.1.0137

[B78] Stocker R. (2012). Marine microbes see a sea of gradients. Science 338, 628–633. 10.1126/science.1208929 23118182

[B79] Sun C.-C. Wang Y.-S. Li Q. P. Yue W.-Z. Wang Y.-T. Sun F.-L. (2012). Distribution characteristics of transparent exopolymer particles in the Pearl River estuary, China. J. Geophys. Res. 117, G00N17 10.1029/2012JG001951

[B80] Vachtman D. Sandler A. Greenbaum N. Herut B. (2013). Dynamics of suspended sediment delivery to the eastern Mediterranean continental shelf. Hydrol. Process. 27, 1105–1116. 10.1002/hyp.9265

[B81] Vardi A. Haramaty L. Van Mooy B. A. Fredricks H. F. Kimmance S. A. Larsen A.. (2012). Host–virus dynamics and subcellular controls of cell fate in a natural coccolithophore population. Proc. Natl. Acad. Sci. U.S.A. 109, 19327–19332. 10.1073/pnas.1208895109 23134731 PMC3511156

[B82] Verdugo P. (2012). Marine microgels. Ann. Rev. Mar. Sci. 4, 375–400. 10.1146/annurev-marine-120709-142759 22457980

[B83] Wetz M. S. Robbins M. C. Paerl H. W. (2009). Transparent exopolymer particles (TEP) in a river-dominated estuary: spatial–temporal distributions and an assessment of controls upon TEP formation. Estuar. Coast. 32, 447–455. 10.1007/s12237-009-9143-2

[B84] Ziervogel K. D'Souza N. Sweet J. Yan B. Passow U. (2014). Natural oil slicks fuel surface water microbial activities in the northern Gulf of Mexico. Front. Microbiol. 5:188. 10.3389/fmicb.2014.00188 24847314 PMC4021148

