# Supplementary material for: Microbial metabolism of transparent exopolymer particles during the summer months along a eutrophic estuary system
Source: Front Microbiol. 2015 May 12;6:403. doi: 10.3389/fmicb.2015.00403 (PMC4436900; doi:10.3389/fmicb.2015.00403)
Supplement: Supplementary file 1 [file DataSheet1.DOCX]

**Supplementary Information**

**Microbial metabolism of transparent exopolymer particles during the summer months along a eutrophic estuary system**

Edo Bar-Zeev^1^* and Eyal Rahav^2^*

^1^ Department of Chemical and Environmental Engineering, Yale University, 9 Hillhouse Avenue, New Haven, CT 06520, USA.

^2^ Israel Oceanographic and Limnological Research, National Institute of Oceanography, Haifa, 8030, Israel.

**

**

**Figure S1.** Relationships between the distance from the coastal water to A) Chlorophyll *a* (Chl *a*), B) TEP concentrations, C) bacterial abundance (BA), D) bacterial production (BP) and E) β-glucosidase (β-glu). Values are average and SD of three sampling dates during the summer months.

**

**

**Figure S2.** Linear correlations between TEP concentrations to A) bacterial abundance (BA) and B) β-glucosidase activity (β-glu). Values are average and SD of three sampling dates during the summer months.
